# Significantly enhanced superconductivity in monolayer FeSe films on SrTiO_3_(001) via metallic δ-doping

**DOI:** 10.1093/nsr/nwad213

**Published:** 2023-08-10

**Authors:** Xiaotong Jiao, Wenfeng Dong, Mingxia Shi, Heng Wang, Cui Ding, Zhongxu Wei, Guanming Gong, Yanan Li, Yuanzhao Li, Binjie Zuo, Jian Wang, Ding Zhang, Minghu Pan, Lili Wang, Qi-Kun Xue

**Affiliations:** State Key Laboratory of Low-Dimensional Quantum Physics, Department of Physics, Tsinghua University, Beijing 100084, China; School of Physics & Information Technology, Shaanxi Normal University, Xi'an 710119, China; State Key Laboratory of Low-Dimensional Quantum Physics, Department of Physics, Tsinghua University, Beijing 100084, China; State Key Laboratory of Low-Dimensional Quantum Physics, Department of Physics, Tsinghua University, Beijing 100084, China; State Key Laboratory of Low-Dimensional Quantum Physics, Department of Physics, Tsinghua University, Beijing 100084, China; State Key Laboratory of Low-Dimensional Quantum Physics, Department of Physics, Tsinghua University, Beijing 100084, China; Beijing Academy of Quantum Information Sciences, Beijing 100193, China; State Key Laboratory of Low-Dimensional Quantum Physics, Department of Physics, Tsinghua University, Beijing 100084, China; Department of Physics, Southern University of Science and Technology, Shenzhen 518055, China; State Key Laboratory of Low-Dimensional Quantum Physics, Department of Physics, Tsinghua University, Beijing 100084, China; International Center for Quantum Materials, School of Physics, Peking University, Beijing 100871, China; Department of Physics, The Pennsylvania State University, University Park, PA 16802, USA; State Key Laboratory of Low-Dimensional Quantum Physics, Department of Physics, Tsinghua University, Beijing 100084, China; State Key Laboratory of Low-Dimensional Quantum Physics, Department of Physics, Tsinghua University, Beijing 100084, China; Beijing Academy of Quantum Information Sciences, Beijing 100193, China; International Center for Quantum Materials, School of Physics, Peking University, Beijing 100871, China; Collaborative Innovation Center of Quantum Matter, Beijing 100871, China; Hefei National Laboratory, Hefei 230088, China; State Key Laboratory of Low-Dimensional Quantum Physics, Department of Physics, Tsinghua University, Beijing 100084, China; School of Physics & Information Technology, Shaanxi Normal University, Xi'an 710119, China; State Key Laboratory of Low-Dimensional Quantum Physics, Department of Physics, Tsinghua University, Beijing 100084, China; State Key Laboratory of Low-Dimensional Quantum Physics, Department of Physics, Tsinghua University, Beijing 100084, China; Beijing Academy of Quantum Information Sciences, Beijing 100193, China; Department of Physics, Southern University of Science and Technology, Shenzhen 518055, China

**Keywords:** monolayer FeSe, interface superconductivity, δ-Doping, zero-resistance temperature, superconductivity transition temperature

## Abstract

Superconductivity transition temperature (*T*_c_) marks the inception of a macroscopic quantum phase-coherent paired state in fermionic systems. For 2D superconductivity, the paired electrons condense into a coherent superfluid state at *T*_c_, which is usually lower than the pairing temperature, between which intrinsic physics including Berezinskii–Kosterlitz–Thouless transition and pseudogap state are hotly debated. In the case of monolayer FeSe superconducting films on SrTiO_3_(001), although the pairing temperature (*T*_p_) is revealed to be 65–83 K by using spectroscopy characterization, the measured zero-resistance temperature (${{T}}_{{\rm c}}^0$) is limited to 20 K. Here, we report significantly enhanced superconductivity in monolayer FeSe films by δ-doping of Eu or Al on SrTiO_3_(001) surface, in which ${{T}}_{{\rm c}}^0$ is enhanced by 12 K with a narrowed transition width Δ*T*_c_ ∼ 8 K, compared with non-doped samples. Using scanning tunneling microscopy/spectroscopy measurements, we demonstrate lowered work function of the δ-doped SrTiO_3_(001) surface and enlarged superconducting gaps in the monolayer FeSe with improved morphology/electronic homogeneity. Our work provides a practical route to enhance 2D superconductivity by using interface engineering.

## Teaser

Enhanced superconductivity with the zero resistance temperature TC0 up to 27.5 K, has been achieved in monolayer FeSe films by ultrathin-d-doping of Eu or Al elements on SrTiO3(001) surface.

## INTRODUCTION

Interface engineering has been proven to be effective in discovering new superconducting systems and promoting superconducting transition temperature (*T*_c_) [1–7]. Monolayer FeSe on SrTiO_3_ (STO) substrates has attracted intense interest owing to its remarkable interface-enhanced superconductivity [8,9]. The interfacial coupling with oxygen-deficient TiO_2_ layer boots pairing gaps up to 17–20 meV [8,10,11], which persisted at >65–83 K (pairing temperature, *T*_p_) [12–15]. However, the hitherto measured zero-resistance temperature $( {T_{\mathrm{c}}^0} )$ by *ex situ* transport is usually ∼20 K [9,16–18] due to multiple reasons. First, monolayer FeSe might be in the crossover regime between Bardeen–Cooper–Schrieffer and Bose–Einstein condensation, where incoherent fermion pre-pairing occurs [15]. Second, nanoscale inhomogeneity, originating from local chemical disorder or spontaneous electronic phase separation from compressible electron confinement by the large dielectric constant of STO [19,20], could widen the transition. The latter is evidenced by the varied gap magnitude from 12 to 17 meV in a couple of millimeters with the gradient distribution of oxygen vacancies (O_v_) spontaneously formed under an electric field [10]. Third, the lattice mismatch and coherent FeSe/TiO_2_ coupling lead to tens-of-nanometer-scaled domains in monolayer FeSe separated by line defects of Fe vacancies [8,21], introducing in-plane electronic confinement in turn. Consequently, the electronic structure and paring strength in the monolayer FeSe vary on the nanometer scale [10,21].

FeSe/TiO_2_ is reminiscent of the built-in multi-interfaces in cuprates (e.g. CuO_2_/LaO) and iron pnictide (e.g. FeAs/LaO) superconductors [22,23], wherein the TiO_2_ layer serves as the charge reservoir layer with intrinsic donors—O_v_ [24]. It is imperative to control the surface O_v_ to improve the interfacial inhomogeneity while maintaining strong FeSe/TiO_2_ interface coupling. However, previous efforts on the FeSe side had little effect: improving the uniformity in the monolayer FeSe is usually at the expense of weakening the interface coupling, or vice versa. For example, post-annealing [8,16,25,26] enhances interface coupling via excess Se desorption but induces high-density line defects. Post-growth Fe deposition improves the homogeneity of monolayer FeSe films with line defects partially replaced by bright boundaries [18,21] but reduces FeSe/TiO_2_ coupling due to the residual interfacial Se atoms.

Within the modulation doping scenario [27], atomically thin doping (δ-doping) has been vastly successful in semiconductor superlattices [28,29]. For TiO_2_-terminated STO(001) surfaces, the deposition of metals with high oxygen affinity could scavenge oxygen, leading to oxygen-deficient TiO_2_ layers [30–32]. Compared with vacuum thermal reduction upon direct current heating, the surface δ-dopants could prohibit spontaneously the electric- and thermal-driven O_v_ clustering. Moreover, increased O_v_ reduces antiferrodistortive rotation in bulk STO [33,34] and hence likely lessens the twin boundaries in the surface TiO_2_ layer. In this regard, pretreatment of STO(001) substrates by δ-doping high-oxygen-affinity metal to introduce surface O_v_ is considered an effective route for both enhancing the FeSe/TiO_2_ interfacial coupling and improving spatial homogeneity.

Here, we perform the δ-doping of Eu or Al atoms on STO(001) substrates before the deposition of the FeSe monolayer. Transport measurements reveal that the superconductivity of monolayer FeSe films grown on the δ-doped STO is remarkably promoted with a $T_{\mathrm{c}}^0$ of 27 K and narrowed transition width Δ*T*_c_ of 8 K, the record high and sharp transition for monolayer FeSe by *ex situ* transport [9]. By utilizing liquid helium temperature (∼4.5 K) scanning tunneling microscopy and spectroscopy (STM/STS), we investigate the morphologies and electronic properties of the Eu-doped and non-doped Nb:STO(001) surfaces and the afterward-deposited monolayer FeSe films. The Eu dopants induced increased O_v_, reduced work functions and lowered the conduction band minimum (CBM) of the Nb:STO(001) surface, accompanied by reduced electronic variation. Monolayer FeSe films on such Eu–Nb:STO(001) exhibit generally enlarged superconducting gaps, indicating the strengthened Cooper pairing with improved electronic homogeneity. Meanwhile, the line defects of Fe vacancies in monolayer FeSe are remarkably reduced and broken into fragments, suppressing the formation of isolated domains and improving spatial homogeneity. We believe that these contribute to the narrowed transition observed in transport measurements.

## RESULTS AND DISCUSSION

### Enhanced superconducting transition of FeSe films on δ-Eu/Al–STO by *ex situ* transport

**Figure 1.**
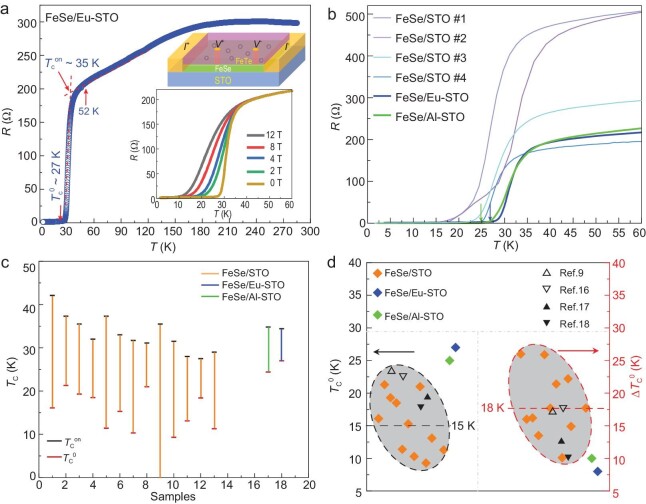
Transport measurements of monolayer FeSe films on Eu-doped/non-doped SrTiO_3_(001) substrates. (a) *R*–*T* curve under zero field for monolayer FeSe/Eu–STO, showing $T_{\mathrm{c}}^{{\mathrm{on}}}$ = 35 K and $T_{\mathrm{c}}^0$ = 27 K. Inset (top): a schematic structure for *ex situ* transport measurements in the heterostructure of FeTe/FeSe/STO. Inset (bottom): *R*–*T* curves under various magnetic fields applied perpendicular to the films. (b) *R*–*T* curves below 60 K showing the superconducting transitions of various samples. (c) The comparison of the superconducting transitions of FeSe on non-doped (13 samples with various growth parameters) and Eu/Al-doped STO substrates. (d) The comparison of $T_{\mathrm{c}}^0$ and Δ*T*_c_ between FeSe/Eu(Al)–STO and FeSe–STO samples. The black and red dashed lines mark the respective average values.

**Figure 2.**
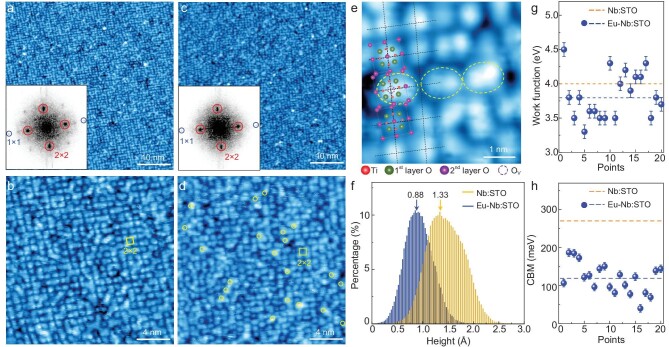
Atomic-scale morphology and electronic structure for Eu-doped/non-doped Nb:SrTiO_3_(001) surfaces. The typical STM topographic images (*V*_s_ = 500 mV, *I* = 50 pA) of Nb:SrTiO_3_(001) surfaces ((a) and (b)) and after 0.03 ML Eu deposited and annealed ((c)–(e)). The insets in (a) and (c) are the corresponding FFT images with red and blue circles marking the (2 × 2) and (1 × 1) Bragg points, respectively. (e) High-resolution morphology images showing O_v_ on (2 × 2) reconstructed surface. The corresponding (2 × 2) structural models with an extra O_v_ are superimposed to show possible oxygen vacancy configuration. Yellow circles in (d) and (e) highlight the possible O_v_. (f) The histograms of apparent height distribution for Eu-doped/non-doped surfaces. (g) Local work function plot deduced from the ln *I*–*z* spectra in SM and Supplementary Fig. S3c. (h) Local CBM values deduced from the d*I*/d*V* spectra in SM and Supplementary Fig. S3d. In (g) and (h), the blue and apricot dashed lines mark the average values for Eu-doped/non-doped surfaces, respectively.

Figure 1a shows the resistance (*R*) versus temperature (*T*) of the FeSe film on Eu-doped intrinsic STO(001) measured at a current of 1 μA, which reveals a superconducting transition from weakly localized metal. The room-temperature-deposited Eu coverage is estimated at 0.03 monolayer (ML), calculated by counting the surface protrusions in morphology images (Supplementary material (SM) and Supplementary Fig. S1). The resistance deviates from the linear extrapolation of the normal state and starts to decrease at ∼52 K and drops completely to zero (defined as resistance within the instrumental resolution of ±0.02 Ω) at $T_{\mathrm{c}}^0\ \sim \ $27 K. By extrapolating both the normal state resistance and the superconducting transition curves, we obtain the intersection as the onset transition temperature $T_{\mathrm{c}}^{{\mathrm{on}}}$ of ∼35 K, which gives a very narrow transition width Δ*T*_c_ of ∼8 K. Notably when defining the onset transition temperature as the temperature at which the resistance deviates from the linear extrapolation of the normal state at 52 K, it reaches the highest value obtained by the same method [9]. The resistivity transition is suppressed under magnetic fields (the inset in Fig. 1a), indicating a typical characteristic of superconductivity. We also conducted δ-doping of Al and observed superconducting transition at a consistent $T_{\mathrm{c}}^{{\mathrm{on}}}$ of 35 K, but slightly lowered $T_{\mathrm{c}}^0$ of 25 K (Fig. 2b and Supplementary Fig. S2), and therefore a little widened Δ*T*_c_ of 10 K at an Al coverage of 0.01 ML.

Figure 1b summarizes the *R*–*T* curves of the δ-doped samples, plotted with those of the other four non-doped samples under similar growth conditions (Supplementary Table S1) for comparison. *R*–*T* curves of those non-doped samples reveal much broader transitions and much lower $T_{\mathrm{c}}^0$. We plot the $T_{\mathrm{c}}^{{\mathrm{on}}}$, $T_{\mathrm{c}}^0$ and Δ*T*_c_ of all samples in Fig. 1c. Clearly, the FeSe films on Eu/Al–STO give the highest $T_{\mathrm{c}}^0$ and the narrowest transition width Δ*T*_c_. Meanwhile, the $T_{\mathrm{c}}^{{\mathrm{on}}}$ of Eu/Al-doped samples remains relatively high and comparable to the average one of FeSe/STO. Further comparing with previously reported *ex situ* transport results of FeSe/STO samples [9,16–18], as summarized in Fig. 1d, the FeSe/Eu–STO samples have an increased $T_{\mathrm{c}}^0$ of almost double the average value (∼15 K) and a narrowed superconducting transition width Δ*T*_c_ of half the average value (∼18 K).

Note, the *in situ* transport reported $T_{\mathrm{c}}^{{\mathrm{on}}}$ = 44 ± 3 K and $T_{\mathrm{c}}^0$ = 29 K [35,36]. Given that the protective FeTe layers for all the *ex situ* transports could reduce the Cooper pairing strength by providing an extra decaying channel for interface phonons [37], we conclude that the interface metallic δ-doping on STO substrate indeed enhances the superconductivity transition in the monolayer FeSe films. On the other hand, despite dopant coverage, the contrasting $T_{\mathrm{c}}^0\ $ values between Eu- and Al-doped samples suggest varied interface modification. The Al element has a higher electronegativity than Eu (2.52 vs. 1.81) [38] and therefore the Al atoms have lower efficiency to combine with oxygen atoms and lower tunability on surface O_v_. Meanwhile, the Al atoms prefer to form larger clusters than Eu, as resolved from the surface morphology image (Supplementary Fig. S1a), which locally enhances the electron scattering and reduces spatial homogeneity as well.

### Microscopic investigation of the δ-doping effect by STM/STS

To understand the δ-doping effect, we performed STM/STS investigation on Eu-doped Nb:STO(001) (0.05 wt%) samples step by step (see SM for the details). After the regular ultra-high vacuum (UHV) annealing, the Nb:STO(001) surface exhibits atomically flat surfaces with (2 × 2) reconstruction, as resolved from the atomically resolved morphology image shown in Fig. 2a and the inserted fast Fourier transform (FFT) image. In the zoomed-in STM image shown in Fig. 2b, periodic (2 × 2) units are clearly identified. After room-temperature deposition, Eu atoms reside on the Nb:STO(001) surfaces as adatoms, with maintained (2 × 2) surface reconstruction, as exemplified in Supplementary Fig. S1b and S1c. To catch the possible surface modification during FeSe deposition, we annealed the Eu-doped Nb:STO(001) at the same temperature as FeSe deposition. As displayed in Fig. 2d and 2e, the surfaces maintain the (2 × 2) reconstruction, except for more bright spots (marked with yellow circles). By overlaying the (2 × 2) structural model with extra O_v_ at the ‘floating’ oxygen site (Fig. 2e), it is found that these defects (dashed purple circles) occur at the bridging sites between two adjacent Ti atoms, consistently with the features of O_v_ previously observed on STO [39] and TiO_2_ [40,41] surfaces. Notably, the intrinsic STO(001) surface applied in transport measurement exhibits $( {\sqrt {13} \times \sqrt {13} } )$ reconstruction. On such surfaces, the room-temperature-deposited Eu resides as scattered adatoms and appears as bright dots upon annealing as well (Supplementary Fig. S1d and S1e). The consistent morphology features indicate that Eu dopants induce similar surface modification on intrinsic STO(001) and Nb:STO(001) surfaces.

To quantitatively estimate the surface modification upon Eu doping, we compare the apparent height, work function and CBM before and after Eu deposition, as plotted in Fig. 2f–h, respectively. The local work function deduced from the formula $\phi = 0.95{( {\frac{{d( {lnI} )}}{{dz}}} )}^2$ was measured by using *lnI–z* spectra and the CBM using d*I/*d*V* spectra (the raw data in Supplementary Fig. S3). After Eu doping, the apparent height peak shifts to 0.88 Å with narrowed width compared with the Gaussian-like distribution centered at 1.33 Å for non-doped ones (Fig. 2f), indicating improved surface homogeneity. The local work function ranges between 3.3 and 4.5 eV with an average value of 3.8 eV (the blue dashed line in Fig. 2g), which is slightly reduced compared with the average value of 4.0 eV (the apricot dashed line in Fig. 2g) for the non-doped surface. More importantly, the surface work functions exhibit much smaller spatial variation than non-doped ones (2.8–4.8 eV, measured by using the same method) [10], indicative of improved electronic uniformity. The average CBM value is 120 meV (the blue dashed line in Fig. 2h), shifted towards *E*_F_, compared with the averaged value of 270 meV (the apricot dashed line in Fig. 2h) for non-doped ones. The reduced surface work function and CBM values upon Eu doping indicate enhanced downward band-bending at the STO(001) surface, resulting from redox reaction due to the higher oxygen affinity of Eu than Ti [38]. Correspondingly, the ratio of Ti^3+^ to the pristine Ti^4+^ increases and the surface TiO_2_ layer becomes further oxygen-deficient, agreeing well with the increased contrast resolved from the atomically resolved images in Fig. 2c–e.

### Morphology and pairing gap of FeSe films by STM/STS

**Figure 3.**
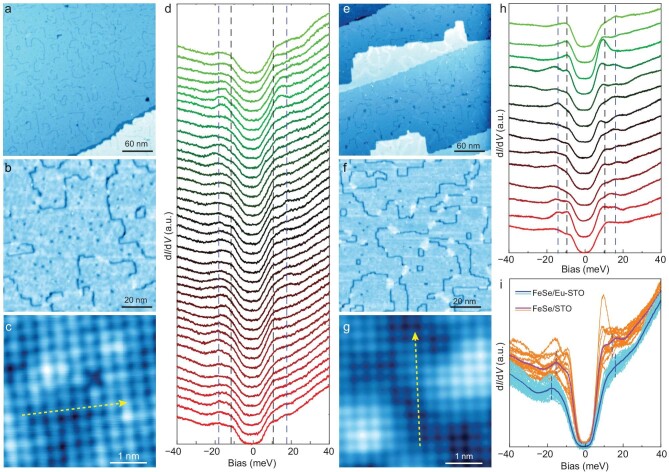
Topographic and spectroscopic comparisons of monolayer FeSe films on Eu-doped/non-doped Nb:STO(001). The typical large-scale ((a) and (e)) and zoomed-in ((b) and (f)) images (*V*_s_ = 1 V, *I* = 50 pA) and atomic resolution image ((c) and (g): *V*_s_ = 50 mV, *I* = 500 pA) of FeSe films on Eu-doped/non-doped Nb:STO(001). (d) and (h) The d*I*/d*V* spectra taken along the yellow dashed line in (c) and (g). The spectra are shifted vertically for clarity. (i) A comparison of d*I*/d*V* curves taken on the monolayer FeSe film on Eu-doped/non-doped Nb:STO(001). The black and blue dashed lines in (d), (h) and (i) are eyes-guided for the coherence peaks of the inner gap and outer gap, respectively.

Figure 3a–c and e–f displays the STM morphology images of the FeSe monolayer films grown on the Eu–Nb:STO(001) and Nb:STO(001) substrates, respectively. Consistently with previous observations [8,21], dark line-like defects are resolved in the large-scale morphology images in Fig. 3a and 3e, while point-like defects from the zoomed-in images on the terrace are shown in Fig. 3b and 3f. The dark line defects correspond to monoatomic chains of Fe vacancies, while the point-like defects can be regarded as the incipient/fragment part of line defects. The average coverage of the dark defects is estimated at ∼0.03 ML for the monolayer FeSe on Eu–Nb:STO, one-quarter less than the ∼0.04 ML for the Nb:STO sample (Supplementary Fig. S4). Correspondingly, the FeSe domains are enlarged in size, irrespective of more incipient point-like defects. Regarding remarkably suppressed superconducting gaps within ∼1 nm of the line defects and the completely vanished superconducting gap in isolated domains of ∼10 nm in diameter formed within looped line defects [21], the broken line defects and reduced closed loops on Eu–Nb:STO(001) correspond to significantly improved spatial homogeneity.

Figure 3d and 3h summarizes the d*I*/d*V* curves taken along the yellow dotted lines in the atomically resolved images in Fig. 3c and 3g, respectively, showing contrasting superconducting gap magnitude and spatial distribution between FeSe/Eu–Nb:STO(001) and FeSe/Nb:STO(001). Consistently with previous reports [8,11], two pairs of coherence peaks are resolved—one pair consistently at ±10 meV and another pair beyond ±15 meV, marked by black and blue dotted lines, respectively. Compared with those on non-doped Nb:STO(001), the pairing gaps for FeSe/Eu–Nb:STO(001) exhibit better spatial uniformity and a little larger outer-gap magnitude (∼18 vs. 15 meV). The contrast can be seen more clearly in Fig. 3i, where the d*I*/d*V* spectra taken on FeSe/Eu–Nb:STO(001) (in blue) and FeSe/Nb:STO(001) (in apricot) are plotted together with the respective averages (in bold) for comparison. The d*I*/d*V* curves of FeSe/Eu–STO universally present larger gap magnitudes and a wider zero-conductance plateau around the *E*_F_, indicating simultaneously enhanced Cooper pairing and improved uniformity.

## DISCUSSION

**Figure 4.**
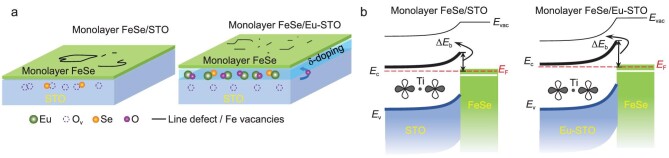
Schematic diagrams for structure and band alignment in FeSe/SrTiO_3_(001) heterostructures without/with Eu dopants. (a) Left/right panel: FeSe/STO heterostructures without/with Eu dopants. (b) Left/right panel: energy band profile across the FeSe/STO heterostructure without/with Eu dopants. The double figure-of-eight patterns represent the O *2p* orbitals. Eu δ-doping lowers the work function and therefore enhances the band-bending (Δ*E*_b_) and the interface charge transfer.

The above spatially resolved spectroscopic characterization of the superconducting gap in monolayer FeSe on Eu-doped Nb:STO(001), together with the transport observation of narrowed superconducting transition on Eu-doped intrinsic STO(001), consistently reveals the simultaneously improved interfacial homogeneity and enhanced interface coupling. This promotion can be primarily attributed to the increased density and improved spatial distribution of O_v_ on the STO(001) surface (Fig. 4a). The Al and Eu atoms with higher oxygen affinity than Ti scavenge oxygen from the STO(001) surface [31,32,38], thus increasing the density of the surface O_v_ but preventing their clustering, as revealed by lowered work functions with reduced spatial variation. According to the interface band-bending scenario, the lower the work function of the STO(001) surface, the stronger the interface charge transfers to the monolayer FeSe (Fig. 4b). Notably, the O_v_ plays essential roles in both the interface charge transfer and the interface electron–phonon coupling [42,43], and the pairing gap of 15–17 meV is well beyond the value of 10–12 meV reached by electron doping [44,45]. Additionally, the dopants help to consume the extra interfacial Se atoms and reduce the interfacial Se–O_v_ combination (Fig. 4a), consequently enhancing electron doping to the monolayer FeSe as well.

The improved morphological homogeneity in the monolayer FeSe films, on the one hand, is verified by the reduction of line defects, especially by suppressing the formation of isolated domains encircled by line defects (Fig. 4a and Supplementary Fig. S4). Notably, the line defects are unique features in the monolayer FeSe that strongly couple to the STO(001) surface [21,46]. Previous study shows that Eu doping could modulate the low-temperature lattice instability of STO [47]. Moreover, antiferrodistortive rotation and ferroelectric distortion both correlate strongly with O_v_ [33,34]. Thus, the δ-doped Eu atoms at least suppress the lattice instability and reduce twin boundaries in the near-surface layers of the STO(001). On the other hand, the δ-doped Eu improves electronic homogeneity on the STO(001) surface down to the atomic scale. The δ-doped Eu atoms could also tune surface chemical potential and reduce the O_v_ clustering, evidenced by reduced surface roughness and narrowed height distribution (Fig. 2g). Combining the strengthened interface effect and improved interface homogeneity, the monolayer FeSe films on the δ-doped STO(001) exhibit enlarged gap magnitude and widened flat zero-conductance around *E*_F_, and therefore sharpened superconducting transition.

The monolayer FeSe films on δ-doped STO(001) still exhibit a *T*_c_ that is much lower than *T*_p_ at ∼65–83 K [12,15] and broader transitions than bulk electron-doped FeSe superconductors [48]. The broad transition has been primarily attributed to the Berezinskii–Kosterlitz–Thouless (BKT) transition in the 2D limit films [9,36]. At the BKT transition temperature *T*_BKT_, the superfluidity disappears with phase correlations changing from a long-range algebraic order to exponential decay via vortex–antivortex unbinding [49]. As consistently confirmed by previous transport measurements [9,36], the *T*_BKT_ (α = 3) values derived from both the power-law $V\sim {I}^{\mathrm{\alpha }}$ dependence and Halperin–Nelson resistivity equation are found to agree well with $T_{\mathrm{c}}^0$, but with wide transitions of a few tenths of *T*_BKT_ due to the size and disorder effect. Given the intense line and point defects and short coherence length [21], the monolayer FeSe films reside in a strongly disordered regime. Under the disorder-tuned superconducting–insulating transition scenario, ubiquitous behaviors of conventional superconductors [50], increasing thickness to the nanometer scale and tuning electronic homogeneity with gating effect are highly applicable to decrease the superconducting transition width [51–55]. In the case of FeSe thin films with an electric double-layer transistor (EDLT), the reported $T_{\mathrm{c}}^{{\mathrm{on}}}$ is up to 50 K and Δ*T*_c_ is down to 4 K [52–54]. It is worth pointing out that the temperature of ∼52 K at which the resistance deviates from the linear extrapolation of the normal state agrees well with a $T_{\mathrm{c}}^{{\mathrm{on}}}$ of ∼50 K in FeSe–EDLT, supporting that the disorder still dominates the wide transition in the monolayer FeSe on the δ-doped STO(001). Moreover, the EDLT doping could enhance electron correlation compared with the monolayer FeSe (3.4 vs. 2.7 m_e_, where m_e_ is the free electron mass) [56,57]. Between *T*_p_ of ∼65–83 K and $T_{\mathrm{c}}^{{\mathrm{on}}}$ of ∼50 K, whether the pseudogap state or BKT physics dominates remains unsolved.

## CONCLUSION

In summary, we employed the δ-doping of metallic Eu/Al on the STO(001) surface to prompt high-dense surface O_v_ with reduced clustering. Transport measurement shows an enhanced superconducting transition with an elevated $T_{\mathrm{c}}^0$ up to 27 K and narrowed transition width Δ*T*_c_ down to 8 K. Microscopic and spectroscopic investigations revealed enhanced interface coupling with improved spatial homogeneity. We anticipate that the superconductivity may be further enhanced under reduced atomic disorders in the interfacial TiO_2_ layer.

## MATERIALS AND METHODS

The details of the experiment are given in the online Supplementary material.

## Supplementary Material

nwad213_Supplemental_File

## Data Availability

The data underlying this article are available in the article and in its online supplementary material.
