# Supplementary material for: Significantly enhanced superconductivity in monolayer FeSe films on SrTiO3(001) via metallic δ-doping
Source: Natl Sci Rev. 2023 Aug 10;11(3):nwad213. doi: 10.1093/nsr/nwad213 (PMC10833465; doi:10.1093/nsr/nwad213)
Supplement: nwad213_Supplemental_File [file nwad213_supplemental_file.docx]

*­Supplementary Information*

**Significantly Enhanced Superconductivity in Monolayer FeSe Films on SrTiO_3_(001) via Metallic δ-Doping**

Xiaotong Jiao, Wenfeng Dong, Mingxia Shi, Heng Wang,Cui Ding, Zhongxu Wei, Guanming Gong, Yanan Li, Yuanzhao Li, Binjie Zuo, Jian Wang, Ding Zhang, Minghu Pan, Lili Wang, and Qi-Kun Xue

**Methods**

**Preparation of SrTiO_3_(001) substrates**

The *ex*-*situ* transport measurements were conducted on intrinsic STO(001) substrates. The intrinsic STO(001) substrates were pretreated with buffered-HCl etching, furnace annealing, and ultrahigh vacuum (UHV) annealing to achieve atomically flat surfaces. The substrates were etched in deionized water (150 °C, 90 min) and 10% HCl solution (room temperature, 45 min), then annealed in a tube furnace under oxygen flow at 980 °C for 3 hours. After being loaded into the UHV molecular beam epitaxy (MBE) chamber, the substrates were degassed at 600 °C for 30 min. After such treatment, the substrates remain bulk insulating and exhibit atomically flat surfaces with $(\sqrt{13}\times\sqrt{13})$ reconstruction.

The LHe-temperature scanning tunneling microscopy/spectroscopy (STM/STS) measurements were conducted on Nb:STO(001)(0.05 wt. %) substrates. After being loaded into the UHV-MBE chamber, the substrates were gradually heated above 1000 °C for 20-30 min, which yield atomically flat surfaces with (2 × 2) reconstruction.

**Eu/Al δ-doping on SrTiO_3_(001)**

Metallic Eu (Al) was deposited from standard Knudsen cells with the source temperature of 350°C for 18 s (870°C for 7 s), while the STO substrates were kept at room temperature. The coverage was estimated at 0.03 ML (0.01 ML) for Eu (Al) by counting the coverage of scattered bright dots in the morphology images.

**Epitaxial growth of FeSe films and FeTe capping layer**

FeSe films were grown by co-evaporating high-purity Fe (99.995%) and Se (99.999%) from standard Knudsen cells at substrate temperatures (*T*_sub_) of 400 °C. The K-cell temperature of Fe (*T*_Fe_) was 980 °C, corresponding to a deposition rate of 0.035 ML per minute. Here, 1 ML is defined as the area density of Fe atoms in a monolayer FeSe film. The K-cell temperature of Se (*T*_Se_) was 92-97 °C, see the details in Table S1. To protect the thin FeSe films from oxidization, FeTe protection layers were grown by co-evaporating Fe and Te (99.9999%) with a flux ratio of ∼1:4 at 270 °C.

|  | Deposition | | | | Annealing | | FeTe (ML) | $T_{c}^{\mathrm{on}}$ (K) | | $T_{c}^{0}$ (K) | Δ*T*_c_ (K) |
| --- | --- | --- | --- | --- | --- | --- | --- | --- | --- | --- | --- |
|  | *T*_Fe_  °C | *T*_Se_  °C | *T*_sub_°C | *t*  min | *T* (°C) | *t* (h) |  | *R*-cross | *R*-drop |  |  |
| **S1** | 980 | 95 | 400 | 23 | 400 | 4 | 10 | 31.7 | 45.0 | 10.3 | 21.4 |
| **S2** | 980 | 94 | 400 | 30 | 400+500 | 0.5+1 | 12 | 37.3 | 46.0 | 11.4 | 25.9 |
| **S3** | 980 | 97 | 400 | 23 | 400 | 12.5 | 10 | 33.0 | 45.0 | 15.3 | 17.7 |
| **S4** | 980 | 95 | 400 | 23 | 400 | 6.5 | 10 | 31.1 | 40.0 | 21.0 | 10.1 |
| **S-Eu** | 980 | 92 | 400 | 65 | 400 | 4.5 | 15 | 34.4 | 52.0 | 27.5 | 6.9 |
| **S-Al** | 980 | 92 | 400 | 50 | 400+430 | 10+4 | 12.5 | 34.8 | 50.0 | 24.4 | 10.4 |

**Table S1.** The growth parameters for selected six samples used in *ex*-*situ* transport measurements, including the samples with Eu/Al doping (S-Eu/S-Al) and four samples without δ-doping (S1-S4). Samples S1-S4 were prepared with almost identical growth parameters as the Eu/Al-doped samples, except for slightly changing *T*_Se_.

**STM/STS measurement**

All the STM/STS data on Nb:STO(001) substrate were collected using a Createc LHe-temperature (ca. 4.5 K) STM system. The morphology images on intrinsic STO(001) (Figs. S1a, S1d, and S1e) were collected using Omicron room-temperature STM. The base pressure is better than 1×10^−10^ Torr. The morphology images were acquired in a constant current mode with a polycrystalline PtIr tip and the bias voltage applied to the sample.

The differential conductance d*I/*d*V* spectra, characterizing the local density of states around the Fermi level, were measured by disabling the feedback circuit, sweeping the sample voltage *V_s_*, and then extracting the differential tunneling current d*I*/d*V* using a standard lock-in technique with a small bias modulation (~1% of the sweeping range) at 937 Hz. The local dI/dV spectra provide a direct measurement of the conduction band minimum (CBM).

To calculate the local tunneling barrier height $\phi=0.95{(\frac{d(\ln I)}{dz})}^{2}$, (*I* in the units of A and z in the units of Å), the values of ln*I* are measured while disabling the feedback circuit and decreasing the tip-sample distance *z*, and then dln*I*/d*z* is extracted from the linear fitting of the ln*I-z* relation.

**Transport measurement**

The schematic for transport measurements is shown in the inset of Fig. 1a. Electrical contacts to the FeSe film through the protective layer were achieved by pressing on the indium electrodes, with ~ 1 mm separation between the two voltage contacts. The transport measurements were performed using the standard four-probe ac lock-in method.

1. **The surface morphologies of Nb:STO(001)/STO(001) after Eu/Al-deposited**

**
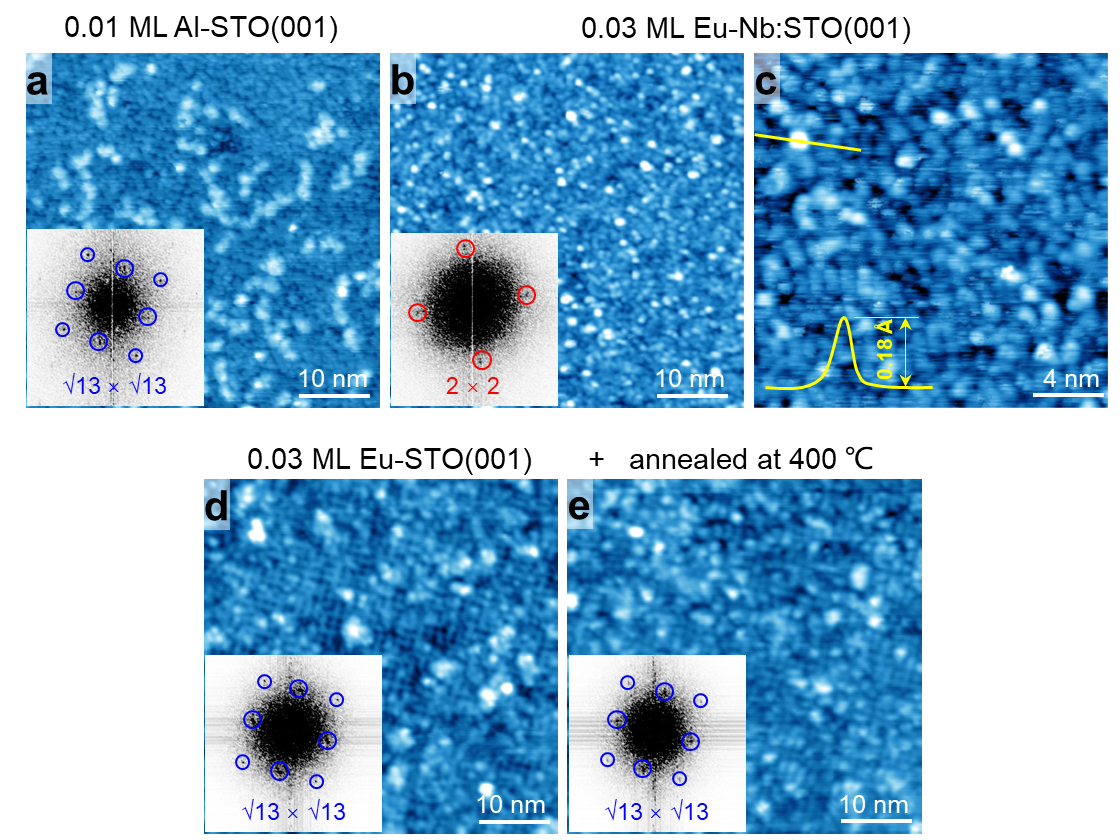
**

**Fig. S1** The typical STM topographic images (V_s_ = 500 mV, I = 50 pA) of Al-Nb:STO(001) (**a**), Eu-Nb:STO(001) (**b**,**c**) and Eu-STO(001) (**d**) and after annealing (**e**). Single Eu-dopant is resolved as bright dots with apparent heights of about 0.18 Å, as shown in the inserted line profile in **c**.

1. **The superconductivity of FeSe/Al-STO(001)**

**
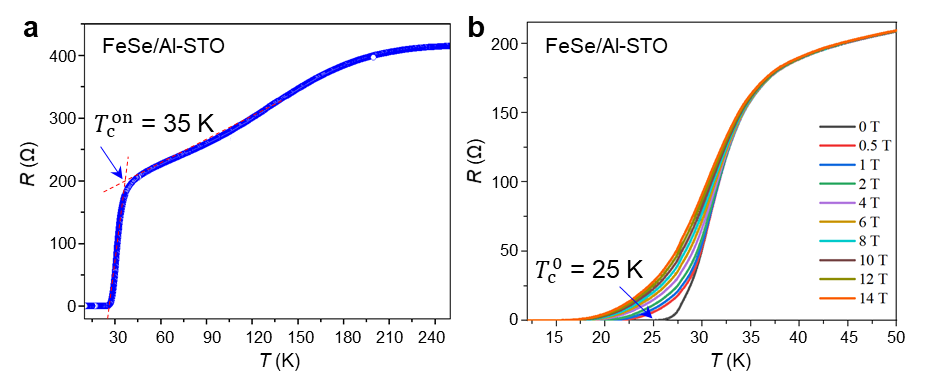
Fig. S2 Transport measurements of monolayer FeSe films on Al-STO(001). a,** The large-scale temperature dependence of resistance under zero field with Al-STO(001). **b,** 𝑅–𝑇 curves at various out-of-plane magnetic fields. Under zero magnetic field, $T_{c}^{\mathrm{on}}$ ~ 35 K and $T_{c}^{0}$ ~ 25 K.

1. **Surface work function and valence state of non-doped and Eu-doped Nb:STO(001)**

**
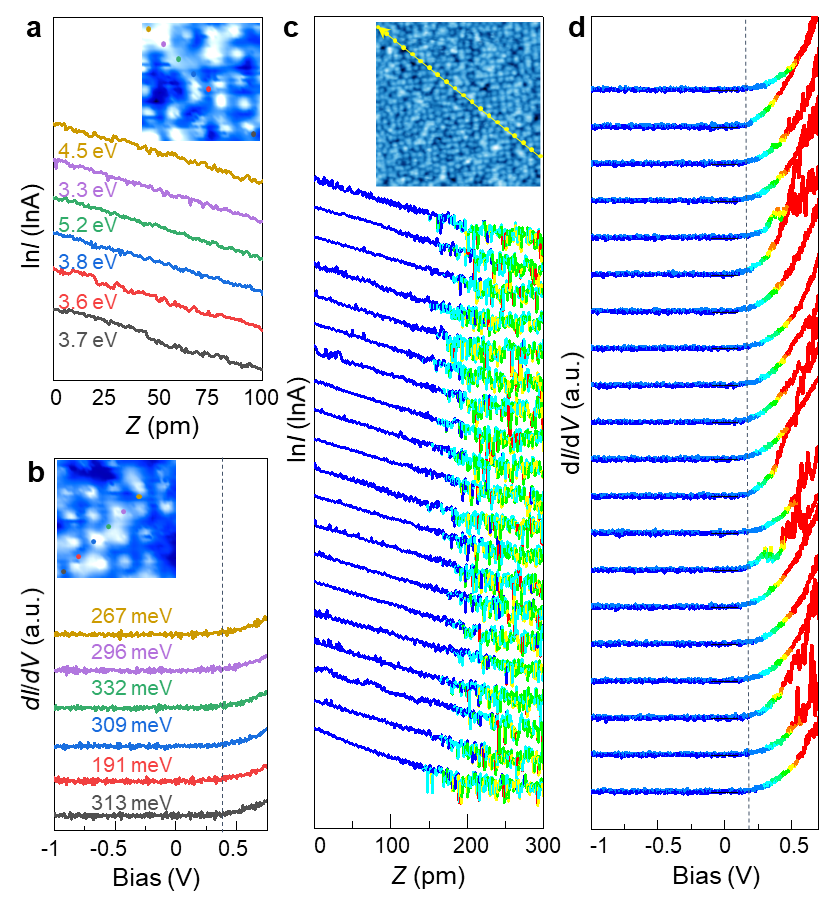
**

**Fig. S3 The ln*I*-*z* and d*I*/d*V* spectra taken on non-doped and Eu-doped Nb:STO(001) surfaces.** **a** the ln*I*-*z* and **b** d*I*/d*V* spectra taken at the marked points in the respective inserted images on non-doped Nb:STO(001) surfaces. c, the atomically resolved image (V_s_ = 50 mV, I = 500 pA) of annealed Eu-Nb:STO(001) surface. **d** the ln*I*-*z* and **e** d*I*/d*V* spectra taken at the marked points in the inserted image in **c**. In **b** and **e**, the d*I*/d*V* spectra show the semiconducting gap with zero density of states (DOS) and the ascending DOS edges. The dashed lines are eye-guided for the DOS edges, i.e., conduction band minimum (CBM).

**4. Comparison of line defects in monolayer FeSe on non-doped and Eu-doped Nb:STO(001)**

**
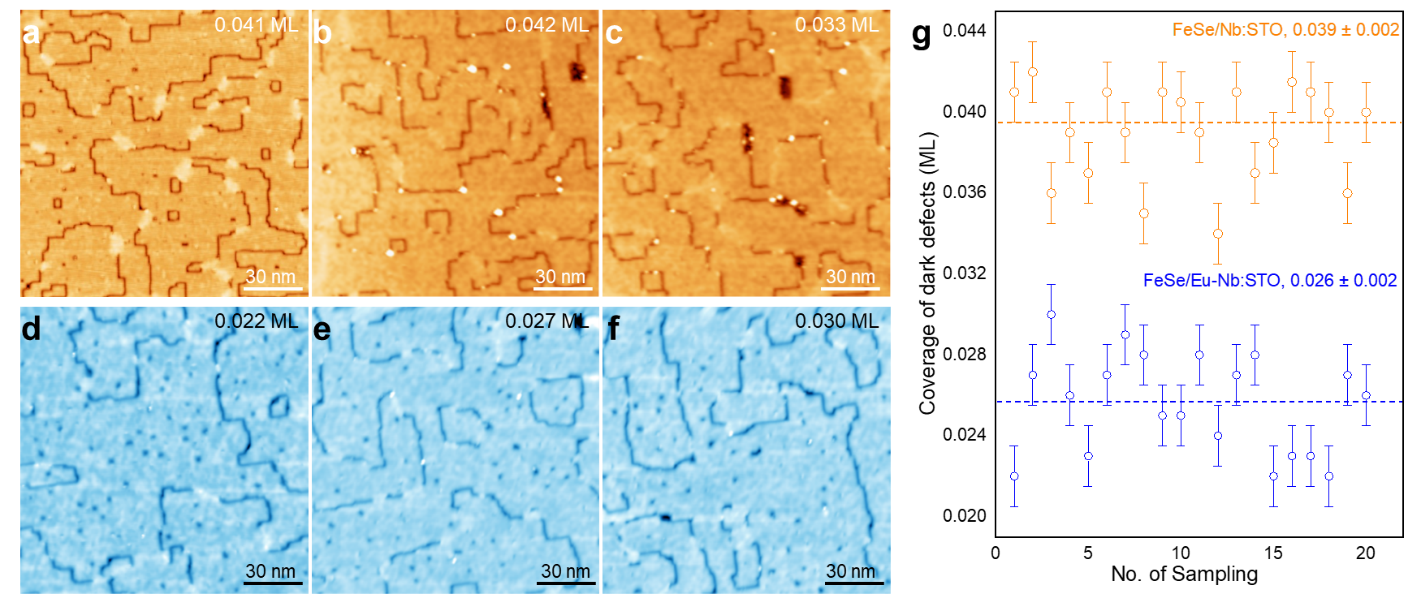
**

**Fig. S4.** **Line/point defects in monolayer FeSe films on non-doped and Eu-doped Nb:STO(001) surfaces. a-c,** Topographic images of FeSe/Nb:STO; **d-f**, Topographic images of FeSe/Eu-Nb:STO (*V*_s_ = 1.0 V, *I* = 50 pA). The coverage of dark defects is labeled in the upper right corner of each image. **g**, The statistics of dark defects from 20 images of different locations. The apricot and blue dashed lines mark the average coverages of 0.026 ± 0.002 ML and 0.039 ± 0.002 ML for FeSe/Eu-Nb:STO and FeSe/Nb:STO, respectively.

**5. Transport results**

**
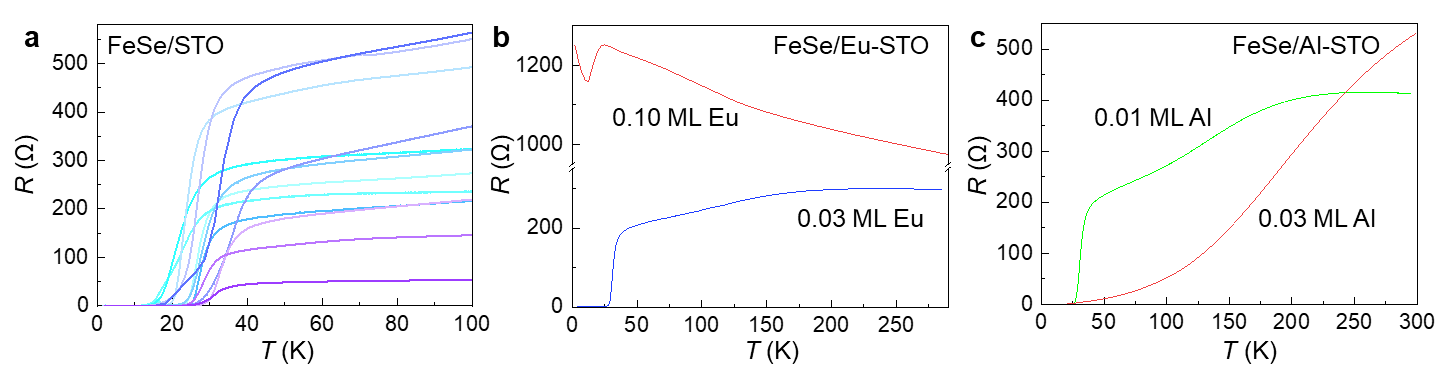
**

**Fig. S5.** ***R*-*T* curves for monolayer FeSe on non-doped and Eu/Al-doped STO.** **a,** *R*-*T* curves for FeSe/STO. **b**, *R*-*T* curves for FeSe/Eu-STO with Eu coverages of 0.10 ML and 0.03 ML. **c**, *R*-*T* curves for FeSe/Al-STO with Al coverages of 0.01 ML and 0.03 ML.
